# The Pharmacological Mechanism of Diabetes Mellitus-Associated Overactive Bladder and Its Treatment with Botulinum Toxin A

**DOI:** 10.3390/toxins12030186

**Published:** 2020-03-16

**Authors:** Chung-Cheng Wang, Yung-Hong Jiang, Hann-Chorng Kuo

**Affiliations:** 1 Department of Urology, En Chu Kong Hospital, New Taipei City 23702, Taiwan; ericwcc@ms27.hinet.net; 2 Department of Biomedical Engineering, Chung Yuan Christian University, Taoyuan City 32023, Taiwan; 3 Department of Urology, Hualien Tzu Chi Hospital, Buddhist Tzu Chi Medical Foundation and Tzu Chi University, Hualien 97002, Taiwan; redeemer1019@yahoo.com.tw

**Keywords:** diabetes mellitus, overactive bladder, inflammation, botulinum toxin

## Abstract

Diabetes mellitus (DM) is an independent risk factor for overactive bladder (OAB). The pathophysiology of DM-associated OAB is multifactorial and time-dependent. Diabetic bladder dysfunction is highly associated with diabetic complications, mainly including diabetic neuropathy and atherosclerosis. Chronic systemic inflammation and bladder urothelial inflammation may contribute to the onset of OAB. Intravesical botulinum toxin A (BoNT-A) injection has proved to be a successful treatment for idiopathic and neurogenic OAB. BoNT-A can inhibit the efferent pathways of the bladder as well as the chronic inflammation and hypersensitivity via the afferent pathways. We conducted a review of the published literature in Pubmed using a combination of two keywords, namely “botulinum toxin A” (BoNT-A) and “overactive bladder”, with or without the additional keywords “detrusor overactivity”, “diabetes mellitus”, “inflammation”, and “urodynamic study”. We also reviewed the experience of our research teams, who have published several studies of the association between DM and OAB. Since limited data support the effectiveness and safety of BoNT-A for treating patients with DM-associated OAB, a comprehensive evaluation of diabetic complications and urodynamic study is needed before treatment. In the future, it is imperative to explore the clinical characteristics and inflammatory biomarkers of diabetes as determining predictors of the treatment efficacy.

## 1. Introduction

Overactive bladder (OAB) and diabetes mellitus (DM) are common health threats and both increase in incidence and prevalence with advancing age. Several epidemiological studies have shown that OAB is more common in patients with type 2 DM than in the general population, and women with DM treated with insulin have higher odds (OR 3.5, 95% CI 1.6–7.9) of urge incontinence than those treated with non-insulin medication [1,2]. A study investigating the prevalence and correlation of urinary incontinence and OAB conducted in Taiwan showed that women who were elderly and menopausal and had a history of DM or hypertension and higher body mass index were significantly predisposed to an OAB [3]. Higher glycosylated hemoglobin levels represented an independent predictor of OAB symptoms among DM patients [4]. Even in early-stage DM, type 2 DM in male patients age <45 years had more OAB symptoms and erectile dysfunction than the controls [5]. Regarding OAB management, a study of 36,560 OAB patients in the US found that patients with DM are more persistent and adherent to OAB medications and have higher odds of filling a second medication prescription than patients without DM [6]. These factors may imply that DM is an important risk factor of OAB, but conventional oral medication is usually not as effective for OAB patients with DM.

We conducted a review of the published literature in Pubmed, using a combination of two keywords, namely “botulinum toxin A” (BoNT-A) and “overactive bladder” with or without the additional keywords “detrusor overactivity”, “diabetes mellitus”, “inflammation”, and “urodynamic study”. We reviewed the pathophysiology of DM-associated OAB, the anti-inflammatory effects of BoNT-A, and the clinical evidence for intravesical BoNT-A injection in patients with DM-associated OAB. We aimed to clarify the role of BoNT-A treatment in these patients.

## 2. Urodynamic Finding in Patients with DM-Associated OAB

Traditionally, diabetic cystopathy is considered as a triad of decreased bladder sensation, increased bladder capacity, and impaired emptying function [7]. Recent clinical and experimental evidence suggests that storage problems such as OAB and detrusor overactivity are common manifestations in early DM. Table 1 summarizes the urodynamic findings of patients with diabetic bladder dysfunction [8,9,10,11,12]. These studies showed that patients (both sexes) with DM had progressive, diverse bladder dysfunction depending on the stage of DM. In addition, diabetic bladder dysfunction is highly associated with other diabetic complications. Majima et al. analyzed the impact of DM on bladder function and found that the presence of both diabetic retinopathy and nephropathy was correlated with the presence of detrusor underactivity [9]. Patients with only diabetic retinopathy had the highest percentage of detrusor hyperactivity and impaired contractility (DHIC). Interestingly, a sub-population of patients reported in our literature search has normal detrusor contractility patterns, but develop detrusor overactivity, which was seen only in cases with neither retinopathy nor diabetic nephropathy. Furthermore, Lee et al. studied urodynamic characteristics and sensory bladder function in type 2 DM women at a mean age of 66.9 years [11]. The electrophysiological evidence indicated an association between impaired A-delta as well as C-fiber bladder afferent pathways and poor emptying function in the women with detrusor underactivity. However, patients with detrusor overactivity had similar current perception threshold values as those in the normal detrusor function group. Ho et al. compared the urodynamic finding in women with DM with and without OAB [13]. Compared to DM without OAB, the women with DM and OAB were more likely to have increased bladder sensation, detrusor overactivity, impaired voiding dysfunction, and a higher percentage of bladder outlet obstruction (BOO). Because of the very different presentations of diabetic bladder dysfunction, we suggest patients with DM-associated OAB undergo a comprehensive evaluation for possible diabetic complications and urodynamic studies before treatment of refractory DM-associated OAB.

## 3. Pathophysiology of DM-Associated OAB

The pathophysiology of DM-associated bladder dysfunction is multifactorial and time-dependent. From experimental and human studies, these changes can be a result of an alteration in the physiology of the detrusor smooth muscle cells, bladder innervation, extracellular matrix, or urothelial dysfunction [14]. In studies of streptozocin (STZ)-induced acute diabetic rats, the up-regulation of M2 and M3 muscarinic biosynthesis in the urinary bladder could lead to increased reactivity to acetylcholine, which results in detrusor overactivity [15,16]. In rats with type 2 DM on a high-fat diet, compared with controls, the diabetic bladders were hypertrophied and had increased volume per void and detrusor muscle contractility to the exogenous addition of carbachol in the compensated stage [17]. Progression from the compensated to decompensated state mainly involves decreased contractility to muscarinic stimulation. In addition, the alternation of the biomechanical behavior of the bladder wall induced by diuresis or diabetes is another important indicator of diabetic bladder dysfunction. In STZ-induced acute diabetic rats, the bladder wall could undergo rapid time-dependent structural and compositional remodeling, mainly including decreased collagen, increased elastin, and a nonlinear stress–strain relationship, and mechanical anisotropy, with greater tissue compliance in the circumferential direction than in the longitudinal direction [18,19].

In a prospective study of 120 type 2 DM patients using simple questionnaires, OAB severity and diabetic peripheral neuropathy were significantly correlated [20]. This finding was similar to that in another study in which the OAB group of women with type 2 DM had a significantly greater mean 5 Hz current perception threshold test value at the big toe compared to diabetic women without OAB [21]. This finding indicated that the hyposensitivity of unmyelinated C fiber afferents at the distal extremities heralded the early stages of diabetic bladder dysfunction. These studies suggest that multiple factors contribute to the occurrence and progression of diabetic bladder dysfunction.

The alternations of the urothelial and underlying lamina propria have been reported that are associated with OAB and diabetic cystopathy. In STZ diabetic rats 9 weeks after onset, scanning electron microscopy showed defective urothelial cells present in the bladders compared with controls, indicating a significant breach of the urothelial barrier [22]. In these diabetic rats, about 20% of the epithelium showed cellular disruption and death within the mucosal lining and umbrella cell loss. In addition, DM had significantly upregulated urothelial gene expression and receptors mainly for glucose metabolism (aldose reductase and sorbitol dehydrogenase), cell survival, cell-signaling receptors (acetylcholine receptors AChR-M2 and AChR-M3, purinergic receptors P2X2 and P2X3), and cell death. The compromised barrier function and alterations in urothelial mechanosensitivity and cell signaling contributed to bladder overactivity.

The findings of the animal studies have been further corroborated by a human study. Bladder mucosa was biopsied from 19 DM-associated OAB patients, 14 OAB patients without DM, and 10 healthy controls [23]. Decreased expression of urothelial junction protein (E-cadherin and ZO-1) and increased urothelial inflammation (mast cells) were noted in the non-diabetic OAB and diabetic OAB patients. The P2X3 protein expression in DM-associated OAB patients was significantly greater than that in OAB patients without DM and controls. However, E-cadherin, mast cells, ZO-1, apoptotic cells, and M2 and M3 muscarinic proteins were comparable between the OAB patients with and without DM. These findings suggest that urothelial dysfunction and chronic urothelial inflammation contribute to the pathogeneses of OAB. However, DM does not aggravate the severity of urothelial inflammation in OAB patients.

## 4. Diabetes and Bladder Inflammation

Chronic inflammation plays a potential role in the pathogenesis of type 2 DM [24]. The possible mechanisms to explain insulin resistance in type 2 DM include oxidative stress, endoplasmic reticulum stress, lipotoxicity, and glucotoxicity. These cellular stresses may induce an inflammatory response or they are exacerbated by inflammation. The vicious cycle of chronic inflammation and related stresses is associated with several diabetic complications, including atherosclerosis, neuropathy, retinopathy, nephropathy, and cystopathy.

Accumulating evidence suggests the roles of several inflammation biomarkers in obesity-induced insulin resistance. Acute-phase proteins such as C-reactive protein (CRP) and pro-inflammatory cytokines (interleukin (IL)-1β and IL-6, and tumor necrosis factor-α), and chemokines are increased in obese and type 2 DM patients, and these markers are reduced when patients are engaged in an intensive lifestyle intervention causing body weight loss [25]. In an 11-year cohort study, the inflammatory biomarkers C-reactive protein and pro-adrenomedullin were independently associated with cardiovascular events and all-cause mortality in type 2 DM patients [26]. Additionally, compared with controls, elevated serum levels of tumor necrotic factor-α and decreased neuregulin-4 (a novel adipokine) were found in diabetic patients and correlated with the severity of diabetic peripheral neuropathy [27,28]. Yeniel et al. assessed atherosclerosis indicators and blood perfusion in the bladder necks in women with OAB. They found that the OAB severity correlated with systemic atherosclerosis and impaired vascular perfusion of the urinary bladder [29]. In diabetic mice, Inouye et al. found that Evans blue extravasation in bladder vessels, an index of peripheral and neurogenic inflammation, correlated with bladder dysfunction [30]. Furthermore, Xiao et al. showed that compared with controls and diuretic groups, diabetic mice have bladders with higher levels of nitrotyrosine (a biomarker of NO-dependent, reactive nitrogen species-induced nitrative stress) and Mn superoxide dismutase (representing the activity of free radical scavengers) [31].

Recently, Hughes Jr. et al. showed that the NLRP3 inflammasome, an intracellular sensor that detects endogenous danger signals and environmental irritants, can sense diabetic metabolites and induce inflammation implicated in diabetic complications and neurodegeneration [32]. Compared to NLRP3 genes of knocked out non-diabetic mice, NLRP3 genes of knocked out diabetic mice had a higher serum glucose level but similar voiding volume, voiding frequency, voiding efficiency, severity of bladder inflammation, bladder Aδ-fibers, and C-fibers density. Interestingly, bladder inflammation and bladder decompression in BOO rats can be inhibited by NLRP3 inhibitor glyburide which might be effective to treat diabetic bladder dysfunction via the similar pathway [33]. In addition, Szasz et al. proposed another possible mechanism of diabetic bladder dysfunction via Toll-like receptor 4 (TLR4) activation. Innate immune system activation via TLR4 leads to inflammation and oxidative stress which causes bladder hypertrophy and hypercontractility [34]. Unlike wild type streptozotocin mice, TLR4 knock out diabetic mice were protected from diabetes-induced bladder dysfunction despite similar levels of hyperglycemia. These evidences, taken together, suggest that inflammatory pathways could be a component of a strategy to prevent or control diabetes and its associated complications.

## 5. Inhibition of Chronic Inflammation and Hypersensitivity by Intravesical Botulinum Toxin A Injection

**Figure 1:**
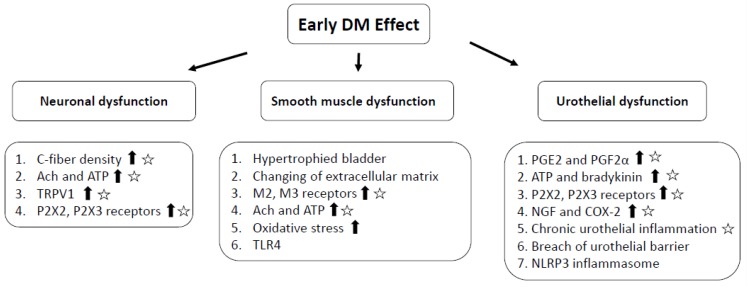
The early effect of diabetes mellitus on the innervation or function of the neuronal component, detrusor smooth muscle, and urothelium. Pentagram sign implies the possible mechanism of BoNT-A to support the clinical efficacy for DM-associated overactive bladder (OAB). The arrow means “increase”.

Injection of botulinum toxin A (BoNT-A) into the detrusor muscle has emerged as a successful treatment for idiopathic and neurogenic detrusor overactivity [35,36]. Figure 1 summarizes some possible mechanisms that have been proposed to support its clinical efficacy for patients with DM-associated OAB [37]. Firstly, BoNT-A is well known for its ability to block the neuronal release of acetylcholine at the neuromuscular junction and therefore to inhibit abnormal smooth muscle contractions. Secondly, BoNT-A not only inhibits the efferent pathway of the bladder but also suppresses hypersensitivity via the afferent pathway. Thirdly, BoNT-A has anti-inflammatory effects and blocks noxious neurotransmitter release from the urothelium, including substance P, calcitonin gene-related peptide, and adenosine triphosphate (ATP). Finally, BoNT-A could be transported both anterogradely and retrogradely along either motor or sensory axons for bi-directional delivery between peripheral tissues or the central nerve system. Significant accumulation of the radio-labeled BoNT-A was noted in the lumbosacral dorsal root ganglia after bladder injection in normal rats [38,39]. Thus, BoNT-A might block not only acetylcholine release from motor nerve terminals but also central synaptic transmission, including glutamate, noradrenaline, dopamine, ATP, gamma-aminobutyric acid, and glycine. Since the pathophysiology of DM-associated OAB consists of afferent and efferent neuropathy, chronic inflammation and urothelial dysfunction, intravesical BoNT-A injection might be effective to treat DM-associated OAB.

Several experimental studies could support the clinical use of BoNT-A in treatment of DM-associated OAB. In acute and chronic inflammation in a rat model, BoNT-A significantly inhibited the release of substance P and calcitonin gene-related peptide after acute and chronic bladder injury [40]. In spinal-cord-injured rats, BoNT-A reversed the ratio of excitatory (ATP) and inhibitory (nitric oxide) urothelial transmitters and decreased non-voiding bladder contraction frequency [41]. In BOO-induced detrusor overactivity in rats, the expressions of nerve growth factor and transient receptor potential vanilloid 1 (TRPV1) proteins in the urothelium were significantly higher in the BOO group than in the control group and the expressions decreased significantly with BoNT-A detrusor injections [42]. In children with neurogenic detrusor overactivity, BoNT-A detrusor injections led to significant reductions in muscarinic M2, M3, P2X2, and purinergic P2X1, P2X2, and P2X3 receptors [43]. In another neurogenic bladder study in 15 children with myelodysplasia, urinary transforming growth factor beta-1 and nerve growth factor declined following intradetrusor BoNT-A injection [44]. These animal and clinical studies of BoNT-A strengthen the evidence of its therapeutic effects in diabetic patients with OAB. Since muscarinic M3 and P2X3 protein expressions in the bladders of DM-associated OAB patients were significantly higher than those in the controls, BoNT-A detrusor injection may provide an alternative treatment for these patients [23].

## 6. Clinical Outcomes of Intravesical Botulinum Toxin A Injection for Patients with DM-Associated OAB

Although numerous laboratory and clinical findings support that BoNT-A inhibits overactivity and chronic inflammation of OAB by different pathways, very few clinical studies have investigated intravesical BoNT-A treatment of patients with DM-associated OAB. Wang et al. reported the first retrospective study to compare the efficacy and safety of intravesical 100 U onabotulinumtoxinA injection in 48 patients with refractory type 2 DM-associated OAB [45]. During the 6-month follow-up period, similar success rates were noted between the diabetic and non-diabetic OAB groups (DM, 56% versus non-DM, 61%, *p* = 0.128). The disappearance rate of detrusor overactivity proved by videourodynamic studies was also similar in both groups (DM, 56.3%, versus non-DM, 47.8%, *p* = 0.41). However, the patients with DM more commonly had adverse events such as large postvoid residual urine volumes and general weakness than the non-DM group.

One hypothesis is that early phase DM causes compensated bladder function, and late-phase DM causes decompensated bladder function [46,47]. Thus, DHIC, a paradoxical condition involving both the storage and voiding phases of bladder function, could happen during the transition from OAB to underactive bladder in DM patients. An interesting study comparing the efficacy and safety of intravesical onabotulinumtoxinA injection in patients with DHIC or OAB showed that the OAB symptoms in both groups significantly improved during the 3-month follow-up period [48]. However, the mean duration of therapeutic efficacy in patients with DHIC was significantly shorter than that of patients with OAB (4.9 ± 4.8 months versus 7.2 ± 3.3 months, *p* = 0.03). Additionally, the incidences of adverse events including acute urinary retention, large postvoid residual urine volume, urinary tract infection, gross hematuria, and general weakness were comparable in both groups.

Furthermore, Kuo et al. analyzed the adverse events after intravesical BoNT-A injection in 217 patients with idiopathic detrusor overactivity [49]. The results showed that male sex, large baseline postvoid residual urine, comorbidities, and higher doses of BoNT-A (>100 U) were risk factors for adverse events after BoNT-A injection for OAB. However, the occurrence of adverse events did not affect treatment outcome. As a result, intravesical BoNT-A injection is still recommended in patients with DM-associated OAB who develop DHIC. Patients should be informed of the possibility of shorter therapeutic duration and adverse events before injection.

## 7. Conclusions

Based on recent basic and clinical studies, intravesical BoNT-A injection appears to be effective and safe in patients with DM-associated OAB. However, this hypothesis requires further validation through randomized controlled clinical studies. A comprehensive evaluation of DM complications and urodynamic studies is needed before BoNT-A treatment for DM-associated OAB to avoid the occurrence of adverse events. Finally, it is important to explore the status of the clinical characteristics and inflammatory biomarkers of DM as determining predictors of BoNT-A treatment efficacy in the future.

## Figures and Tables

**Table 1:** Summary of urodynamic findings in patients with diabetes.

Author [reference]	Patients (*n*)	Mean Age (years)	DM Duration (years)	DO	DHIC	DU	Normal	SUI
Majima [9]	57M	65.8	10	5 (9%)	18 (32%)	22 (39%)	12 (23%)	NA
Karoli [8]	44F	54.8	11.6	10 (23%)	NA	5 (11%)	9 (16%)	22 (48%)
Bansal [10]	52M	61.3	11	20 (39%)	NA	41 (79%)	NA	NA
Gali [12]	21M + 19F	64.5	10.9	7 (18%)	24 (60%)	4 (10%)	5 (13%)	NA
Lee [11]	86F	66.9	11.4	12 (14%)	NA	30 (35%)	33 (38%)	NA

DO: detrusor overactivity; DHIC: detrusor hyperactivity and impaired contractility; DU: detrusor underactivity; SUI: stress urinary incontinence; NA: not available.
